# Identifying technology clusters based on automated patent landscaping

**DOI:** 10.1371/journal.pone.0295587

**Published:** 2023-12-14

**Authors:** Bergeaud Antonin, Verluise Cyril

**Affiliations:** 1 HEC Paris, Paris, France; 2 Centre for Economic Policy, London, United Kingdom; 3 Center for Economic and Policy Research, Paris, France; 4 Paris School of Economics, Paris, France; 5 College de France, Paris, France; Abu Dhabi University, UNITED ARAB EMIRATES

## Abstract

We introduce a new general methodological approach for accurately and consistently retrieving a large set of patents related to specific technologies. We build upon the automated patent landscaping algorithm by incorporating a tractable amount of human supervision to improve the accuracy and consistency of our results. We demonstrate the efficacy of our approach by applying it to six novel and representative technologies: additive manufacturing, blockchain, computer vision, genome editing, hydrogen storage, and self-driving vehicles.

## 1 Introduction

Modern growth theory underscores the pivotal influence of frontier technologies in fostering long-term economic advancement and broader societal progression [1, 2]. The genesis, dissemination, and adoption of these technologies is a central subject of investigations spanning various field of the social sciences, including policy, finance, education, and management [3–6]. Yet, grasping the diffusion of frontier technology is empirically challenging, given the intricacies involved in accurately characterizing and delineating them. This paper presents a novel approach based on patent data to tackle this challenge.

Many scholars and analysts employ patent data to measure innovation. Traditionally, they have turned to technological classifications designated by intellectual property (IP) offices, such as the “Cooperative Patent Classification” (CPC), to identify clusters of patents related to given technologies. However, this method has notable limitations, an issue often referred to as the “patent classification problem” [7]. Standard classifications by IP offices are constructed with technical attributes in mind and frequently deviate from an economist’s interpretation of a technology. Designed primarily for engineers, these classifications emphasize techniques over functional applications. As a result, a single technology can be fragmented across multiple, seemingly unrelated classes. For example, [8] points out that a subclass related to dispensing solids included patents on both manure spreaders and toothpaste tubes.

To address this issue, we introduce a novel, generalizable method to accurately and consistently retrieve a vast set of patents related to a specific technology, in the sense of a functional application. Enhancing the machine learning-based algorithm proposed by [9]–which emulates human curation on a large scale–we infuse a tractable measure of human supervision to elevate the accuracy and uniformity of our outcomes. To illustrate our method, we apply it to six cutting-edge technologies: additive manufacturing, blockchain, computer vision, genome editing, hydrogen storage, and self-driving vehicles. Selected to span various economic sectors and maintain conceptual cohesion, these technologies showcase the versatility of our approach. Critically, our methodology is adaptable, allowing researchers to assess any technology using patent data and trace its evolution over time.

Delineating technologies within the vast patent corpus is a topic of active exploration. Scholars such as [10, 11] have delved deep into the patent corpus, examining the diffusion of frontier technologies characterized by their radicalness, novelty, speed of diffusion, potential for significant impact, and associated risks [12]. On the other hand, focused studies like [13] specifically probe phenomena such as the rise in software patenting. Regional patent offices also contribute by proposing mapping of technologies, often based on experts judgement [14]. Our research aims to provide additional depth to the current discourse on methodologies used to categorize patents within certain technological domains. In pioneering efforts like [15]’s examination of Computed Tomography Scanners, the patent classification leaned heavily on manual curation–a process that was undeniably tedious and time-intensive. Some researchers, including [10], favored rule-based categorizations, marrying keywords with CPC classes. However, these approaches often rested on *ad hoc* rules, necessitating deep domain expertise. The European Patent Office’s investigation into automated vehicles serves as a salient example of this rule-based methodology [16].

Recent advancements have increasingly leaned towards scalable, technology-driven techniques in patent categorization. Notably, [9] introduced the innovative concept of “automated patent landscaping”, which closely emulates the precision of manual classification while only needing a select subset of representative patents. This movement towards automation has been bolstered by the incorporation of natural language processing and machine learning to enhance the patent categorization process. A noteworthy example is the Fung Institute’s initiative, which leverages machine learning for automatic patent labeling. Complementing this, [17] refined the [9] technique to cater specifically to AI-related patents. Our contribution situates itself in this evolving landscape. Drawing inspiration from the latest research, we have augmented the algorithm from [9]. We have modified both the selection mechanism for the representative set of patents and the algorithm’s subsequent expansion from this foundational set. In essence, our methodology combines a small amount of human work and automated landscaping to select patents related to a given technology with a high degree of precision and with no limitation in geographical coverage, and has been designed with the view of being easily extended to other technologies.

## 2 Technology definition and selection

We illustrate our methodology using six example frontier technologies. Although our approach can be adapted to a multitude of cases, we selected these six candidates because they span various fields, are universally acknowledged for their high potential, and can be distinctly defined by a specific set of tasks.

### 2.1 Definition

Technology is a widely used term and can refer to many different concepts. In the economic and innovation literature, we classified its main usages into three categories which we refer to as “technique”, “functional application” and “application field”. A *technique* is a set of processes sharing a common methodological paradigm. Two distinct techniques can share a common goal. For example, TALENs, Zinc Fingers and CRISPR are all distinct techniques pursuing the same goal of editing the genome. A *functional application* is a high level goal which is directly targeted by one or several techniques in the course of their developments. Examples include computer vision and genome editing. The range of their market applications can vary and usually exceed a single market. Eventually, an *application field* is an existing or newly created economic market which can leverage functional application to develop new or improve existing products. Examples of application fields include smartphones, nuclear power generation, etc…

In our approach, we want to work at the *functional application* level. This comes as a natural choice since we are interested in frontier innovation which has the potential to give advanced economies a significant growth momentum. Hence, our focus is on technologies which, like General Purpose Technologies, have the ability to infuse progress in a large range of applications. Function applications can be characterized by a set of tasks (for example, one of the task of autonomous vehicles is to enable cars to make autonomous decisions) which we will use to guide our selection procedure.

### 2.2 Selection

There are two main ways to define a set of technologies of interest: the supervised and unsupervised approaches. The most common approach, the “supervised”, is based on human curation of technology-related documents. This is the approach followed by [10] who define a list of technologies in the high-tech segment from prior knowledge. The second and more recent approach, the “unsupervised”, combines text mining (specifically “topic modelling” techniques) and technology-related corpus to identify technologies (e.g. topics) without any use of prior knowledge. Such a method is implemented by [11] who use earnings conference call transcripts to uncover technologies which are the most frequently cited for their contribution to companies’ momentum (see also [18] for an application to scientific fields).

Although extremely appealing, the unsupervised approach presents two limitations in our context. First, and most importantly, relying on past financial and corporate documents will invariably miss frontier technologies with still nascent market applications. Second, existing topic modeling techniques cannot guarantee that the identified “topics” (here technologies) are conceptually homogeneous. Without any supervision, selected technologies might (and will) include techniques, functional applications and application fields indifferently.

We opted for the supervised approach but designed a methodology to minimize our own biases and discipline the selection process. In particular, we sought to restrict to technologies that are considered as impactful and radical by many different institutions of different nature and geographical location. Do to so, we first screened a large number of reports and articles published at different time and dedicated to breakthrough technologies. These articles have various sources: international institutions ([19–21]), national agencies ([22, 23]), industry associations [24], experts ([25]) and consulting companies ([26, 27]). We took care to include sources from both developed and developing countries. From those documents, we listed without any *a priori* more than 30 technologies in a broad sense. Then we classified these items into the three aforementioned categories (technique, functional application and application field) and kept only those entering the “functional application” category. Eventually, we reviewed the remaining candidates (goals, recent breakthroughs, expected economic impact, and development stage) with two main objectives in mind: 1) only keep technologies that have already proven to have market applications or are expected to do so in the near future and 2) cover a large number of distinct application fields. From our initial list of technologies, we ended up with six frontier technologies: additive manufacturing, blockchain, computer vision, genome editing, hydrogen storage and self-driving vehicles. See S1 Appendix for more details about how we selected the six technologies.

### 2.3 Six different technologies

Before moving to the description of the automated patent landscaping methodology, we briefly discuss the characteristics of the six technologies considered in this article and why they constitute a relevant panorama of frontier technologies at the dawn of the 21^*st*^ century. A brief individual description and discussions about market potential are available in S1 Appendix.

Additive manufacturing, blockchain, computer vision, genome editing, hydrogen storage, and self-driving vehicles are all technologies that are seen as having the potential to fundamentally disrupt our daily lives, are growing rapidly, and are receiving large investments. They are however at different stage of their development. Additive manufacturing, and computer vision are technologies that have been developed for decades with existing commercial applications. It is usually acknowledged that the first 3D-printing patents are filed in the first half of the 1980s [28] and the history of computer vision starts with the development of digital image scanner in the 1960s. Self-driving vehicles have been the subject of significant research at least since the 1970s, but the process of developing a fully autonomous commercial vehicle is not yet complete. Finally, hydrogen storage, genome editing and blockchain are more recent technologies, even if in some case, research started many years ago. S2 Fig shows the number of patent publications in each of these technologies each year (these patents have been selected with a methodology that we detail in the next section from the Google Patent dataset).

These technologies also differ in their development. While Additive manufacturing, computer vision and self-driving vehicles are the subject of massive investments by large industrial groups for several years, startups play a big role in pushing the blockchain technologies which is very recent and allows firms to scale-up without the need of massive investment in tangible capital. The development of genome editing technologies remains closely linked to university laboratories, with an important coordination effort (see e.g. [29]). Using a simple classifier based on the name of the assignee and which can be found here (see also the corresponding repository) we find that in 2019 about 12% of patents in genome editing are filed by a university of a public research institution. This number is below 5% in all other five technologies.

Last but not least, these six technologies have applications (or potential applications) in a wide varieties of sectors. Additive manufacturing is already adopted in many different industrial sectors, blockchain has implication in data processing but also in finance, computer vision is an important brick of the development of AI systems, genome editing is mostly concentrated in the pharmaceutical sector, hydrogen storage in energy and self-driving vehicle in transport.

## 3 Materials and methods

### 3.1 Automated patent landscaping with humans in the loop

In this section, we introduce automated patent landscaping, how it relates with existing approaches in economics, what are its limitations and how we address them.

#### 3.1.1 The traditional approach

Determining the scope and boundaries of a technology using the patent corpus, or organizing patents into clusters, has been a longstanding challenge. A variety of methods have been attempted in order to address this issue. The three main tools that have been utilized are technological classifications, citations, and keywords. While each of these tools can provide useful insights, they are also prone to introducing a significant amount of noise and variability into the analysis. In this section, we provide qualitative intuitions on these limitations. Section 3 will further quantify them. Technological classes are based on technical principles which are only partially related to the concept of technology we are looking for (functional application). Citations between patents have clear limitations in this case as well. Patent-to-patent citations are generated in order to define the scope of the technological monopoly granted to the patentees and to assess the validity of a patent over prior art. Proximity in the sense of functional application is then just one of the many reasons to generate a citation. Besides, the network of citations is very sparse and a large number of patents are never cited [30]. Finally, keywords can help identify patents dealing with a technology. However, language is highly variational: there are many ways to mention the same idea and at the same time a given word can have many different meanings. Hence, one can expect neither comprehensiveness nor accuracy from keywords alone. In this context, following [15], manual patent curation might appear to be the most accurate way to delineate a technology in the patent corpus.

#### 3.1.2 Automated patent landscaping

That is where the *automated* patent landscaping introduced recently by [9] makes an important contribution. The authors develop a *semi-supervised* machine learning framework to emulate human-made technology classification. The algorithm only requires a small set of patents as input—the *seed*—which must be representative of the technology of interest. The algorithm then *expands* to “likely related” patents using both technological classes and citations (forward and backward). Specifically, it first expands to technological classes which are overrepresented in the seed and then expands twice on citations. Importantly, at this stage, we know that the resulting expansion set includes patents unrelated to the target technology or “false positives”. The false positives are then *pruned* out using a classification model, based namely on the patent abstract, applied to the expansion set.

More precisely, the classification model is trained to distinguish between patents that belongs to the seed and a set of patents randomly drawn from the universe of patents, outside the expansion set (so-called *anti-seed*) and therefore “likely unrelated” to the target technology. This approach ultimately returns a group of patents in the target technology at virtually no cost, except for the curation of the seed patents. Importantly, no human intervention is needed to elaborate the set of rules determining whether a patent belongs or not to the target technology: semantic patterns are learned from the data.

The approach described in [9] has already demonstrated a high level of potential, but it still exhibits certain limitations that are worth noting.

First, the pruning model is trained on “polar” cases while we would prefer to apply it to “intermediary” cases. The seed patents (positive examples) are selected to be at the “core” of the target technology. On the contrary, anti-seed patents (negative examples) are chosen from the complementary of the expansion set, hence potentially very far away from the target technology. For example, when trying to select patents related to the blockchain technology, the anti-seed might contain patents on drugs, car engines and semi-conductors. Hence, even if the algorithm performs well on the validation set, it is not necessarily indicative of its performance when applied to patents in the expansion set, which may contain a significant proportion of “intermediary” examples. These examples may not be directly related to the target technology, but are still relatively close to it in terms of their characteristics or features. Training the model using a large majority of polar cases may therefore affect the overall validity of the classification model and the performance of the algorithm.

Second, the algorithm does not adequately consider the effect of variations in the data, such as the impact of changes to the seed data on the algorithm’s output. The robustness of the algorithm, or its ability to produce consistent results despite variations in the input data, is an important factor to consider when evaluating the reliability of the results and the overall interpretation of the analysis. Robustness is a crucial aspect to consider when assessing the confidence we can place in the results and the conclusions that can be drawn from them.

#### 3.1.3 A new extended approach

Our extended approach aims to address these two limitations. Firstly, we *augment* the anti-seed with more challenging examples. These complex examples naturally emerge from the human labeling of the seed patents tailored for each technology. We begin by reviewing existing efforts in the literature to landscape our target technologies using conventional methods. Specifically, we draw from this literature and their stipulated selection criteria (often based on technological classes and/or keywords; see, for example, [31–33] for Blockchain–a comprehensive list of our sources is provided in S2–2 in S2 Appendix). This guides us in generating a set of indicative patents, keywords, and CPC classes to seed for each technology. S2–3 in S2 Appendix elaborates on these criteria for each technology. Leveraging these guidelines, we randomly sample a set of potential candidate patents and manually inspect them by perusing their titles and abstracts, labeling them as either relevant to the technology or not (refer to S2–1 Table in S2 Appendix). Crucially, we retain the patents we exclude as they offer invaluable “hard examples”. Even though these patents met one or more criteria set by prior landscaping attempts, human scrutiny based on abstracts deemed them irrelevant. These typically represent the “intermediate” examples we intend our classification model to learn from, ultimately differentiating them from patents genuinely related to our target technology. We term this collection of examples the *augmented anti-seed*. Ultimately, our model is trained using both the traditional anti-seed as proposed by [9] and our augmented anti-seed to formulate the negative samples. S2 Appendix delves deeper into our seed construction process.

Second, we address the data variation question by implementing a series of robustness tests based on random variations in the seed. Specifically, we investigate how variations in the seed affect the expansion and the pruning outcomes. Formally, to test the robustness of the expansion, we draw random subsets from the seed, run the expansion using each of these subsets and compare the generated expansion sets. Next, we assess the pruning robustness by iterating over various random train-test splits of the annotated data. Various models are trained on varying sets of training data for each technology. Pruning robustness is ultimately evaluated by looking at models’ agreement on a sample of out-of-training patents. Detailed results are reported in Section 3.

## 4 Results and discussion

In this section we go through the main steps of the actual deployment of the algorithm and we show that our results, in addition to being accurate and consistent, also exhibit patterns in line with technology experts’ expectations.

### 4.1 Algorithm deployment

To begin with, it is important to note that contrary to [9], we deploy the algorithm at patent family level rather than at patent publication level. A patent family is a collection of patent documents that are considered to cover a single invention in the sense that they share the same priority claims. Their technical contents are identical. Hence, considering only one document per family does not imply any loss of information while significantly reducing the total number of items considered. We start from the Google Patent dataset which counts around 120 million patent publications and 70 million patent families. Using patent family rather than publication has two important practical advantages. First, it enables us to consider all families with at least one publication having a known English abstract. That way, we ultimately cover more than 86% of all publications since 1970, while only 76% of patent publications do have a non-null abstract in our database. Detailed coverage is reported in S1 Fig for the main patent offices. Second, it minimizes the amount of texts to be classified at the pruning stage. Each family is processed only once, even if it includes more than one patent. This improves the overall computational tractability of the algorithm. Each individual patent then inherits from the characteristics of its family.

#### 4.1.1 Construction of the seed

Next, we delve into the algorithm deployment itself. As already discussed in Section 1, our work starts one step before the algorithm described by [9]. This first step consists in the definition of rules to identify a set of candidates. These candidates are picked out of patents which match at least one of the rules that we were able to find in the specialized literature. These rules include technological classes, keywords and patent similarity. Indeed, there are instances in the specialized literature where specific patents are identified as being particularly representative or significant for a particular technology. In our study, we used the patents most similar to these key patents as defined in the Google Patents database and expand our dataset to include these related patents. A random set of candidates are then labeled by humans based on the abstract and detailed annotation guidelines (see S2–1 Table in S2 Appendix). Annotation guidelines guarantee both transparency and replicability. In practice, we labeled candidates until at least 300 were accepted, forming the technology *seed*. Notably, the rule-based candidates consistently contained a significant proportion of false positives, which we excluded. This excluded set, termed the *augmented anti-seed*, contains in particular patents that would have been mistakenly included in the target technology set if only a simple rule-based approach had been applied.

#### 4.1.2 Expansion

Starting from the seed, the following step is the expansion. Regarding this step, we mainly follow to the [9]’s procedure. We first expand to technological classes that were over-represented in the seed and then expand twice using citations (backward and forward). Note however that we had to adapt at the margin to take into account our choice to work at family level rather than publication level. In particular, we expressed citations in terms of the patent family rather than the usual publication format. For each family, we considered all citations received (forward) and sent (backward) by any patent in that family.

#### 4.1.3 Pruning

Finally, our pruning stage also differs from [9] along 3 dimensions. First comes the composition of the training data. As already discussed, we add an augmented anti-seed to the seed and anti-seed described in their paper. Second, while our predecessors used not only text but also citations and technological classes as input to the classification model, we only restricted to text. In our view, both technological classes and citations imply potential pitfalls at this stage. Using technological classes in both the expansion and the classification model can generate pathological cases. Assuming that all technological classes in the seed are found important, then the anti-seed and the seed have no technological class in common which makes the classification task trivial. Regarding citations, by construction, patents in the second level of the citation expansion (L2) have no citations in common with the seed. Hence, considering citations in the classification task implies a systematic and uncontrolled bias against patents in the part of the expansion which we find undesirable. Third comes the model itself. We implement 3 different neural network architectures popular for text classification tasks: the multi-layer perceptron (MLP), the convolutional neural network (CNN) and a transformer, specifically a pre-trained Bert encoder. We provide an overview of these architectures in the following sub-section. The actual pruning is performed using the Transformer model which exhibits both the highest performance and consistency.

### 4.2 Performance and consistency

The most simple architecture we consider is the multi-layer perceptron (MLP). This architecture can be seen as a stack of logistic regressions and treats tokens or groups of tokens independently. Although it can be successful at identifying key phrases, it is unable to handle context and might eventually be seen as a sophisticated phrase matcher. We then turn to a second model and implement a Convolutional Neural Network (CNN). This architecture leverages the sequential nature of text through the use of feature maps (masks). These feature maps are there to detect sequences of tokens with a common and discriminant “meaning”. CNN performances usually dominate those of MLP models thanks to this enriched understanding of language. However, they lack “memory” and cannot handle long context as feature maps typically focus on 3 to 5 token-long spans of text. Finally, we consider the Transformer architecture which was recently introduced [34] and has achieved spectacular results in many natural language processing (NLP) tasks, including text classification. Transformers rely on a core mechanism called *attention* which enables them to “understand” tokens in the context of neighboring tokens. Transformers are very large models trained at masked language completion on very large texts and eventually fine-tuned on specific tasks (e.g. text classification). This pre-training allows downstream users to start from a model that already embodies a large “understanding” of language. A limited number of examples is then enough to adjust weights and achieve high performances on more specific tasks in specific contexts. This is especially well-suited when annotating examples is costly. The main drawback of using Transformers is their high computational costs: transformers are almost intractable using traditional Central Processing Unit (CPU) and require Graphics Processing Unit (GPU).

#### 4.2.1 Performance

We then train all these models. The task is a standard binary text classification. Specifically, we train and evaluate each model on ten distinct train-test sets for each technology. We implement this approach as a cross-validation method to have an estimate of the impact of random variations of the training set on both the performance of the model and its out of (training) sample predictions—later called *consistency*. Let us first focus on performance before moving to consistency later. We report the median *precision*, *recall* and *F1-score* for each technology and model architecture in Table 1. These metrics were all computed on the test set, that is, on examples not used to train the model. The precision is the share of texts that the model assigns to the seed and which are indeed part of it. The recall is the share of texts in the seed which were indeed predicted to be part of it. The F1-score is the arithmetic mean of the precision and recall. We observe that MLP and CNN architectures tend to exhibit similar F1-score. However, MLP models have higher precision and lower recall than CNN. This relates to the fundamental nature of MLP. As stated earlier, MLP can be seen as a sophisticated keyphrase matcher which usually has high precision but low recall. In any case, the transformer outperforms both of the models and achieves around 90% of median F1-score for all technologies except for self-driving vehicles (79%). This latter technology is indeed harder to classify even for humans. The very same technology can be used to automate driving or to assist human driving. In the former case, we would accept a patent while in the latter it would be rejected. In the rest of the paper, we will use results from this latter model.

**Table 1:** Models performance.

	MLP			CNN			TRF		
	P	R	F1	P	R	F1	P	R	F1
**Additive Manufacturing**	0.89	0.79	0.84	0.79	0.85	0.81	0.86	0.92	0.89
**Blockchain**	0.90	0.81	0.86	0.83	0.88	0.86	0.97	0.98	0.97
**Computer Vision**	0.89	0.81	0.85	0.86	0.87	0.87	0.87	0.95	0.90
**Genome Editing**	0.89	0.87	0.88	0.87	0.91	0.88	0.86	0.94	0.89
**Hydrogen Storage**	0.86	0.73	0.80	0.76	0.83	0.78	0.92	0.98	0.93
**Self-driving Vehicle**	0.79	0.65	0.71	0.69	0.73	0.71	0.75	0.85	0.79

Reported performance metrics were computed on the test set—unseen during training. Performance metrics are reported as follows: P for precision, R for recall and F1 for F1-score.

#### 4.2.2 Consistency

As already discussed, although performance *per se* matters, it is also crucial to understand how variations in the seed data can affect the results of the algorithm. We identify two channels. First, data variations can affect the expansion. The latter depends on the seed and has a critical role. It determines the set of documents which will be considered by the pruning model. Second, data variations can affect the pruning itself. The pruning model depends on the seed, the anti-seed and the augmented anti-seed and ultimately determines which documents in the expansion are to enter the technology or not. Robustness to random variations in the data is then crucial to ensure that algorithm results can be exploited rigorously. To investigate the consistency of the expansion, we generate random subsets of the seed. Specifically, we consider 3 different sizes: 90%, 70% and 50% of the initial seed and draw 10 subsets for each size. We then proceed to the full expansion starting from these distinct seeds and compute the pairwise family overlap of the generated expansion sets for each technology and seed size. Detailed results are reported in Table 2. We find that the average pairwise family overlap exceeds 89% in all cases. This remarkably high number indicates a high level of consistency for the expansion step and reassure regarding the relevant of the delimited technology.

**Table 2:** Median pairwise expansions overlap.

	90%	70%	50%
**Additive manufacturing**	0.99	0.93	0.89
**Blockchain**	0.99	0.98	0.96
**Computer vision**	0.99	0.96	0.92
**Genome editing**	0.99	0.99	0.98
**Hydrogen storage**	0.99	0.97	0.95
**Self-driving vehicle**	0.99	0.97	0.95

For each size (90%, 70% and 50%), we drew 10 random subsets of the seed and proceeded to an expansion. For each pair, we computed the share of families in the two expansions. We report the median share of overlapping families across all expansion pairs.

Next, we looked at how the pruning stage is affected by variations in the training data. As discussed above, we trained the same architectures on 10 different train-test splits (of respective size 80%-20%) for each technology as a way to emulate natural variations in the data. We then apply these models to a set of 10,000 out-of-training-sample documents randomly drawn from the expansion. For each technology, we then look at the standard deviation of the ten scores (each score ranging between 0 and 1) for each document and report its median in Table 3. We find that the standard deviation of the predicted scores is usually very low, most of the time below 0.05 which supports the consistency of the pruning step.

**Table 3:** Models robustness (Median dispersion in predicted scores).

	MLP	CNN	TRF
**Additive manufacturing**	0.029	0.082	0.017
**Blockchain**	0.008	0.047	0.003
**Computer vision**	0.015	0.029	0.010
**Genome editing**	0.003	0.001	0.004
**Hydrogen storage**	0.015	0.037	0.005
**Self-driving vehicle**	0.039	0.091	0.011

For each model architecture, we trained 10 models using distinct random subsets (80%) of the training set. Each model was then applied to a set of 10,000 texts (out of training set). We report the median standard deviation (at the sample level) of the predicted scores across models.

To summarize, our evaluation of the performance and consistency of the extended patent landscaping is very encouraging. In the next section, we take a first look at the set of patents that constitute each of the six technologies and consider the external validity of our approach.

### 4.3 External validation

We now use the output of the algorithm to investigate whether our results make sense. To do so, we first consider the top assignees and top inventors as reflected by the total number of patents they hold. To identify individuals and firms, we used the harmonized name of assignees and inventors from the IFI CLAIMS dataset (available through Google Patent public data). It is however important to note that this harmonization does not always guarantee that two different names of the same entities are actually merged in the same entity (e.g. Toyota Motor Co Ltd and Toyota Motor Corps). We do it for each studied technology and then confront these results with prior insights from technology-specialized literature as well as background checks. These lists of top assignees and inventors are reassuringly consistent with our priors and existing information. They also provide insights about the main actors of the different technologies considered. Finally, we also use the PatCit dataset [35] and look at the top 3 most cited academic articles by patents in each technology.

#### 4.3.1 Top 10 assignees by technology

Top panel of Table 4 reports the top 10 assignees for each technology by the number of patents they were granted worldwide.

**Table 4:** Top 10 assignees and top 10 inventors.

	Additive manufacturing	Blockchain	Computer vision	Genome editing	Hydrogen storage	Self driving vehicle
**Top assignees**						
**1**	Samsung Electronics Co Ltd	Alibaba Group Holding Ltd	Canon KK	Univ California	Toyota Motor Co Ltd	Toyota Motor Co Ltd
**2**	Hewlett Packard Development Co	IBM	Sony Corp	Pioneer Hi Bred Int	Honda Motor Co Ltd	Bosch Gmbh Robert
**3**	Xerox Corp	Qualcomm Inc	Samsung Electronics Co Ltd	Du Pont	Nissan Motor	Honda Motor Co Ltd
**4**	Asml Netherlands BV	Samsung Electronics Co Ltd	Koninkl Philips Electronics NV	Regeneron Pharma	Toyota Motor Corp	Nissan Motor
**5**	Gen Electric	LG Electronics Inc	Matsushita Electric Ind Co Ltd	Genentech Inc	Matsushita Electric Ind Co Ltd	Ford Global Tech LLC
**6**	Eastman Kodak Co	Sony Corp	Sharp KK	Monsanto Technology LLC	Sanyo Electric Co	Denso Corp
**7**	Canon KK	NChain Holdings Ltd	Seiko Epson Corp	Harvard College	Hyundai Motor Co Ltd	Toyota Motor Corp
**8**	Fujifilm Corp	Huawei Tech Co Ltd	Lg Electronics Inc	Hoffmann La Roche	Air Liquide	Hyundai Motor Co Ltd
**9**	Siemens AG	Intel Corp	Qualcomm Inc	Univ Pennsylvania	Panasonic Corp	Mitsubishi Electric Corp
**10**	IBM	Ericsson Telefon Ab L M	IBM	Centre Nat Rech Scient	GM Global Tech Operations Inc	Bayerische Motoren Werke AG
**Top inventors**						
**1**	Silverbrook Kia	Karczewicz Marta	Karczewicz Marta	Murphy Andrew J.	Ovshinsky Stanford R.	Tabata Atsushi
**2**	Lapstun Paul	Zhang Li	Zhang Li	Macdonald Lynn	Ukai Kunihiro	Shimizu Yasuo
**3**	Ng Hou T.	Zhang Kai	Nishi Takahiro	Mcswiggen James	Edlund David J.	Nordbruch Stefan
**4**	Vermeersch Joan	Wright Craig Steven	Kondo Tetsujiro	Zhang Feng	Fetcenko Michael A.	Hayakawa Yasuhisa
**5**	Van Damme Marc	Qiu Honglin	Wang Ye-kui	Rosen Craig A.	Taguchi Kiyoshi	Lynam Niall R.
**6**	Lewis Thomas E.	Yang Xinying	Chen Ying	Stevens Sean	Wakita Hidenobu	Watanabe Kazuya
**7**	Zhao Lihua	Wang Yue	Chen Jianle	Ruben Steven M	Maenishi Akira	Yasui Yoshiyuki
**8**	Patibandla Nag B.	Liu Hongbin	Yamazaki Shunpei	Wilson James M.	Young Kwo	Liu Jun
**9**	Ganapathiappan Sivapackia	Wang Zongyou	Kadono Shinya	Ni Jian	Nishio Koji	Breed David S.
**10**	Ye Jun	Fukushima Shigeru	Sugio Toshiyasu	Gurer Cagan	Reichman Benjamin	Matsuno Koji

Assignees and inventors are ranked based on the total number of patents for each technology over the whole corpus of patents. The harmonization of assignees’ names is taken from the CLAIMS dataset.

A first observation is that most of the obvious players in each technology are present. For the sake of brevity, we focus on some remarkable high-ranked agents for each technology and explain why they were indeed expected. Starting with additive manufacturing, Xerox and Hewlett-Packard are two large companies that traditionally developed printers and which naturally moved to 3D printing technologies. In the field of blockchain, Alibaba, Intel, nChain and IBM are also in the top list of assignees in the expert-based landscaping of blockchain innovation proposed by [32]. The most prolific assignees in the field of Computer vision include firms that build and sell electronic devices, including cameras (Canon, Sony etc…). Interestingly, the top assignees in the field of genome editing are universities such as University of California Berkeley, Harvard University and University of Pennsylvania. As explained in Section 1.3, this technology as the characteristics of being very tightly connected to the academic world and breakthrough advances have been made in the laboratories of famous universities. Nevertheless, the list also reports large companies that develop chemistry and pharmaceutical products like Regeneron and Dupont. Overall, these findings are consistent with results from an overview of patenting in the genome editing technology field proposed by [36]. The field of hydrogen storage technologies is primarily dominated by car manufacturers, reflecting the primary use of this technology to power hydrogen-propelled vehicles. Similarly, the realm of self-driving cars features many traditional car manufacturers, notably including Toyota and Ford, both of which actively publicize their advancements in autonomous vehicle development. The roster of leading assignees also features automotive equipment suppliers like Bosch and Denso Corp. Notably, Toyota, Ford, and Bosch are highlighted as top assignees in the field according to Chapter 3 of [37].

On top of very large firms that spread over a large number of different technologies such as IBM and Samsung, we also note the presence of a number of firms that are much more specialized in a specific field. This is notably the case of Air Liquide for hydrogen storage, nChain for blockchain, ASML for additive manufacturing, Regeneron pharma for genome editing and Denso Corp for self-driving cars.

#### 4.3.2 Top 10 inventors

Moving from firms to people, the bottom panel of Table 4 reports the top 10 inventors for each technology by the number of patents they were granted worldwide.

As previously, for the sake of brevity we focus on the most emblematic and high-ranked inventors. We can note the presence of Marta Karczewicz in both Blockchain and Computer Vision. M. Karczewicz is a prolific inventor working at Qualcomm Technologies, Inc.. She is famous for having developed many technologies related to data compression which facilitates the transfer of important mass of information. The methods she developed are very central for many computer-related technologies such as computer vision and blockchain. As a recognition for her contributions, the EPO named her one of the three finalists for the award of European inventor of the year 2019 [38]. Considering additive manufacturing, the most prolific inventor in the field is Kia Silverbrook. He is also a famous inventor who holds more than 9,000 patents worldwide [39]. K. Silverbrook founded Silverbrook Research, a company that developed digital printing and 3D printing technologies, among other inventions. In the field of genome editing, our top inventor is Andrew Murphy. He is the vice president in charge of research of Regeneron, a biotechnology company that develops different drugs and recently made important progress in new therapies using CRISPR [40]. We also note the presence of Feng Zhang, a Professor at MIT and researcher at the Broad Institute. He is well known for his role in the development of optogenetics and CRISPR. He is also famous for his ongoing patent dispute with Chemistry Nobel Prize recipients J. Doudna and E. Charpentier over CRISPR-cas9 human application priority. Next, regarding hydrogen storage, Stanford R. Ovshinsky was a prolific inventor and engineer who contributed enormously to various fields, including energy science, and own hundreds of patents. In particular, he developed solid hydrogen storage technologies and founded the company Ovshinsky Innovation LLC at the end of his life to continue to explore alternative sources of power. Finally, in self-driving vehicle technology, Atsushi Tabata is an engineer at Toyota who published several articles related to the automation of driving controls.

#### 4.3.3 Top academic publications

As a last exercise, we use the PatCit database [35, 41] to look at the most cited academic papers by technology. PatCit is a tool that lists all citations from patents to research articles (also known as Non Patent Literature citations) that were used as a source. We report these articles along with the corresponding journal title in Table 5. To save space, we only report the top 3 for each technology but a longer list of doi is available in Table S3–2 in S3 Appendix. As expected, the most cited articles, i.e. those that were the most pivotal in producing the ideas used in the development of the patents of each technology, are published in journal that are related to the technology. These journal can have a direct and clear link, for example, the International Journal of Hydrogen Energy is mentioned for hydrogen storage and the Proceedings Eighth IEEE International Conference on Computer Vision for computer vision.

**Table 5:** 3 most cited articles by technology.

	Title and Journal
**Additive Manufacturing**	
1	Immersion lithography at 157 nm
	*Journal of Vacuum Science & Technology B*
2	Diaryliodonium Salts. A New Class of Photoinitiators for Cationic Polymerization
	*Macromolecules*
3	Intelligent paper
	*Electronic Publishing, Artistic Imaging, and Digital Typography*
**Blockchain**	
1	The design and implementation of a log-structured file system
	*ACM SIGOPS Operating Systems Review*
2	Scale and performance in a distributed file system
	*ACM SIGOPS Operating Systems Review*
3	A case for redundant arrays of inexpensive disks (RAID)
	*Proceedings of the 1988 ACM SIGMOD international conference on Management of data*
**Computer Vision**	
1	Overview of the H.264/AVC video coding standard
	*IEEE Transactions on Circuits and Systems for Video Technology*
2	Rapid object detection using a boosted cascade of simple features
	*Proceedings of the 2001 IEEE Computer Society Conference*
3	Robust real-time face detection
	*Proceedings Eighth IEEE International Conference on Computer Vision*
**Genome Editing**	
1	Duplexes of 21-nucleotide RNAs mediate RNA interference in cultured mammalian cells
	*Nature*
2	Functional anatomy of siRNAs for mediating efficient RNAi in Drosophila melanogaster embryo lysate
	*The EMBO Journal*
3	RNA interference is mediated by 21- and 22-nucleotide RNAs
	*Genes & Development*
**Hydrogen Storage**	
1	Compact methanol reformer test for fuel-cell powered light-duty vehicles
	*Journal of Power Sources*
2	Steam reforming of natural gas with integrated hydrogen separation for hydrogen production
	*Chemical Engineering & Technology*
3	A safe, portable, hydrogen gas generator using aqueous borohydride solution and Ru catalyst
	*International Journal of Hydrogen Energy*
**Self-driving Vehicles**	
1	Adaptive Cruise Control System Aspects and Development Trends
	*SAE Technical Paper Series*
2	Atmospheric CO2 Content in the Past Deduced from Ice-Core Analyses
	*Annals of Glaciology*
3	Advanced public transport information in Munich
	*International Conference on Public Transport Electronic Systems*

Top 3 academic papers cited in the patents in each of the six technologies on the front page retrieved using PatCit [35].

However, the links may also seem less obvious, reflecting the complexity of externalities from academic research to the development of innovations. For example, the second most cited article for self-driving vehicle is a 1982 research that discusses CO2 concentration in the atmosphere. Since one of the goals of autonomous cars is to reduce the carbon footprint of transportation, this topic is often mentioned and discussed in the relevant patents.

### 4.4 Discussion

#### 4.4.1 Comparison with other approaches

Using our candidate annotation exercise, we can compare those results with the performance that would have been obtained based on the rules used by existing attempts to landscape our six technologies of interest. Specifically, it enables us to obtain performance metrics for rule-based approaches using technological class, keywords and patent similarity. Although our approach does not enable us to compute all the performance metrics reported before, we can compute the precision of simpler approaches (for example using only a set of relevant keywords). We find that rule based candidate selection delivers both low and variable precision performances across technologies. Specifically, precision from CPC-class rule-based patent selection ranges from 0.01 (blockchain) to 0.34 (additive manufacturing). Precision from keyword rule-based selection goes from 0.09 (blockchain) to 0.89 (genome editing) for an average of 0.32. Precision from patent similarity ranges from 0.02 (additive manufacturing) to 0.57 (genome editing). All these metrics are reported in S3–1 in S3 Appendix. It clearly appears that our approach to delineate technologies from the corpus of patents not only achieves good performance but also outperforms traditional rule-based methods. Hence, the set of patents selected using this new approach is both more precise and more complete than those of most existing attempts.

Our approach to constructing the seed and anti-seed for patent classification, though more labor-intensive, offers clear advantages. By meticulously designing the seed and anti-seed, we ensure that our classifier focuses on discriminating between closely related cases, enhancing precision in complex scenarios. This contrasts with methods such as those by [9], which are trained on more distinct, polar cases. Our advanced transformer-based architecture further supports this nuanced approach, enabling more effective and efficient handling of large patent datasets. The comprehensive effort in seed construction, while demanding, facilitates a robust and transparent classification framework, effectively addressing potential biases and ensuring balanced classifications. As such, while our methodology may not lend itself to direct performance comparisons with existing rule-based approaches due to its distinct foundational principles, it represents a significant advancement in the field of automated patent classification, providing a more accurate and comprehensive understanding of technology landscapes.

#### 4.4.2 Use and misuse of this classification

Our methodology offers flexibility in extending to various technologies, providing researchers a robust framework to delineate technologies through meticulous seed construction, adhering to our “automated with human in the loop” approach. Key to this extension is the precise definition of the set of tasks and stringent rules for patent inclusion in the seed, as these are crucial for maintaining the output’s quality. Specifically, it necessitates selecting technologies characterized by well-defined functional applications (e.g. solar photovoltaic materials, hardware for quantum computing, biodegradable plastic, battery technology for electric vehicle etc…). While our approach is versatile, we recommend caution when applying it to broad and multifaceted concepts such as AI or green innovation. These terms often encompass a diverse range of notions, which might challenge the specificity required for our methodology. However, specific technologies within these broader domains can certainly be effectively delineated using our approach. This recommendation is aimed at preserving the accuracy and relevance of the classification, ensuring that the methodology is applied where it can be most effective and meaningful.

#### 4.4.3 Ethical concerns

In the context of automated patent classification, it is essential to address associated ethical considerations. Our methodology integrates both automated processes and human oversight. This integration aims to rectify potential biases that might arise from exclusive reliance on automation, ensuring a more robust and transparent classification framework. By utilizing a diverse and representative training dataset, we actively mitigate the risk of unbalanced classifications, which could inadvertently misguide research and development trajectories. Moreover, our approach remains consistent with the public domain status of patents, circumventing potential privacy complications. Thus, our methodology offers not just efficiency in patent landscaping but also a conscious alignment with ethical best practices in academic research.

## 5 Conclusion

In this study, we have refined and built upon the methodology introduced by [9], facilitating the reliable differentiation of frontier technologies within the global patent corpus. Our method’s efficacy stems from its precision, adaptability, and consistent results across diverse technological domains and requires only a minor expert judgment to generate a relevant seed.

Our approach could hold value for researchers and analysts seeking to understand the contributions of various stakeholders–whether nations, institutional groups, or individual firms–within specific technological arenas. For those intent on pinpointing major contributors to nascent technologies or tracking developmental trajectories, our methodology provides a systematic and effective framework for such analyses.

## Supporting information

S1 Appendix Selection of technology. (PDF)

S2 Appendix Construction of the seed. (PDF)

S3 Appendix Additional results. (PDF)

S1 Fig Share of patent families with an abstract in English. (PDF)

S2 Fig Number of patent publications by technology. (PDF)
