# Supplementary material for: Identifying technology clusters based on automated patent landscaping
Source: PLoS One. 2023 Dec 14;18(12):e0295587. doi: 10.1371/journal.pone.0295587 (PMC10721033; doi:10.1371/journal.pone.0295587)
Supplement: S1 Appendix — (PDF) [file pone.0295587.s001.pdf]

S1 Selection of technologies

S1.1 Choice criteria

The term "technology" is ambiguous. It is used in many contexts to refer to distinct concepts. To make sure that we adopt a consistent approach, it's key to clearly distinguish between these concepts.

- **Technique:** We call *technique* a set of processes sharing a common methodological paradigm. Importantly, two distinct techniques differing by the methods involved can still share the same goals. E.g. statistical learning, deep learning, fuzzy logic for Natural Language Processing; TALENs, Crispr, Zinc fingers for genome editing; etc.
- **Functional application:** We call *functional application* a high level goal which are directly targeted by one or more techniques in the course of their development. They are not necessarily related to immediate market outcomes and the range of their market applications can vary. E.g. Computer vision, Natural Language Processing, Cultured meat, 3D printing, Genome editing, Bio plastic, etc.
- **Application field:** We call application field an existing or newly created economic sector which can leverage functional application to develop new/improve existing goods and services. E.g. Agriculture, Telecommunication, Transportation, etc. In some cases, the application field can be confounded with a family of *devices/goods/services* (e.g. smartphone)

We are interested in the so-called "breakthrough technologies". Using the above framework, breakthrough technologies correspond to *functional applications* which are expected to have a large *impact* on one or more application fields. Many *techniques* might be competing to become dominant at this functional application.

To select candidate technologies, we refer to various sources listing the potential technologies of the 21th century, while keeping in mind that we need a technology to have the following features:

- Advancing rapidly or experiencing breakthrough that drive accelerated rates of change or discontinuous capability improvements
- Having a potential broad impact, i.e. touching various companies and industries and affecting (or giving rise to) a wide range of machines, products, or services.
- Having a high economic impact
- Being potentially disruptive, i.e. able to transform how people live and work, create new opportunities and businesses
- Being sufficiently discussed in the expert literature so we can access existing attempt at landscaping the technology

- Being sufficiently "new" to ensure that we do not consider technologies whose advanced is well planned and the results of an industry consensus (in particular the 4G, 5G, 6G communication protocol).

Our initial list of technologies is presented below along with the type of technology (A for application field, F for functional application, T for technique).

- **Computer Science:** Quantum computing (F/T), Blockchain (F), Three dimensional chip (T), Application-specific integrated circuit (T), Neuromorphic chips (T), Grid computing (T), Cloud computing (T), Field Programmable Gated Array (T), Edge computing (T)
- **Biotechnoloy:** Genome engineering (F), Personalized medicine (F), mRNA vaccines (F/T)
- **Information & Communication:** Internet of Things (F), Mega constellation (F), 5G/6G (T)
- **Energy:** Smart grid (F), Wind energy (F), Solar energy (F), Marine & Tidal energy (F), Internet of energy (F), Hydrogen storage (F), Fusion Power (T), Hydrogen battery (T), Advanced energy storage (T), Organic solar cell (T)
- **Transportation:** Self driving vehicles (F), Drones (F), Electric vehicle (A/F)
- **Agriculture:** Cultured meat (F), Vertical farming (F)
- **Materials:** Bioplastic (F), Additive manufacturing (F), Graphene material (T), Carbon nanotubes material (T)
- **Human-machine interface:** Exoskeleton (F), Brain computer interface (F)
- **Artificial Intelligence:** Computer vision (F), Natural Language processing (F), Speech processing (F), Machine translation (F)

A first selection based on selecting only functional application and removing technologies that are either at a too early or uncertain stage or without enough documentations in terms of patent landscaping boils down to the following list: hydrogen energy storage, blockchain, genome engineering, cultured meat, additive manufacturing, computer vision, natural language processing. While all these technologies would fit our criteria, we further reduce this list to 6 technologies. We remove natural language processing and cultured meat. The former because we already included computer vision as a technology within AI and the latter because the existing patent landscaping documents did not allow us to define a clear frontier.

S1.2 Description of the selected technologies

S1.2.1 Additive Manufacturing

A brief description Additive manufacturing, or 3D printing, is the construction of a three-dimensional object from a Computer-aided Design (CAD) model or a digital 3D model. Contrary to standard manufacturing techniques, additive manufacturing does not start from an

existing block that would be cut and shaped but builds from a raw material, layer to layer. The very concept of 3D printing appeared in the 1950s (then called molecular spray) and the first patents filed are usually dated in the 1970s (depending on the sources, either by Charles W. Hull or by Johannes F Gootwald and in 1974 the term of 3D printing was coined in the New Scientist.

The term 3D priting encompass a large variety of underlying printing methods, the most commonly used being known as Fused deposition modeling (or FDM) uses a continuous filament of a thermoplastic that is directed by a head to create the desired shape. Among its advantages, 3D priting generates little waste and allows more customization and flexibility in creating complex shapes.

3D printing is still predominantly used in prototyping (40%) and some small and large scale finished goods production (30%) as well as research and education purposes (10%) in various sectors in particular automotive, aerospace and machine industry [1]. While the technology is already well diffused in the industry, several challenges remain. First the cost of material is 10 to 200 more expensive than their non-printing equivalent. In addition, 3D printing is still too slow compared to other prototyping technologies. Second, there is an important need to extend the ability of current 3D printers to support more than 1 material at a time. Third, investment are needed to improve 3D printing of metallic device.

See [2] for more details.

Market potential In 2019, estimates of the additive manufacturing market is estimated at $10.9 worldwide [1]. While it represents only 1% of valued added in manufacturing at this date, it could go up to 5% as the tech further mature. Many different industry are likely to adopt this process, from textile and in particular sportswear, aicraft and aerospace manufacturers to the design of very specific medical-device. Not surprisingly, its growth rate is expected to reach up to 20 percent per year during the next decade.

S1.2.2 Blockchain

A brief description Blockchain is a distributed database (or ledger) shared across a public or private network. Each computer of the network gets a copy of the full ledger as a way to prevent system failure. The database itself is a growing list of records (called blocks) linked together using cryptography. Each block contains: i) a cryptographic hash of the previous block, ii) a timestamp and iii) transaction data. Consensus and or validation protocols are used to validate a new block before it can be added to the chain. This prevents fraud without the need of a central authority.

The development of blockchain is tied to the Bitcoin, but does not limit to the support of crypto-currencies. Indeed, the range of applications or potential application of this technology is very large (financial transactions more generally, but also any type of record and verification system such as patents, land titles etc...). Fundamentally, blockchain can be viewed as a way to ensure transactions in a broad sense in a low-trust environment without the need of a supervising actor.

The technology has been developed since the 1990s but experienced several breakthrough since the 2010s. In 2012, [3] introduced the proof of stake which might be used as a replacement

of the proof of work used, for example, as part of the bitcoin blockchain. The proof of stake overcomes a major limitation of early versions of the blockchain: energy consumption (due to many miners performing the same operation). It is notably used by the crypto-currency Eutherium.

In 2014, the Ethereum's white paper described Bitcoin as a weak version of smart contract - a transaction protocol intended to automatically execute, control or document legally relevant events and actions according to the terms of a contract or an agreement. Although smart contracts were first proposed in the early 1990s by Nick Szabo, envisioning blockchain as a support for smart contract in general considerably widens its potential impact and fields of applications by ascertaining trust between unknown parties.

See [4, 5] for more details.

Market potential It is still difficult to assess the size of the market for blockchain. Some estimates suggest that the growth rate of total sales from blockchain could reach 50% per year and reach more than $40 billion by 2027. In any case, according to [6], the potential developments of blockchain are very pervasive and broad and are likely to represent several billion in investment.

S1.2.3 Computer Vision

A brief description Computer vision aims to give computers the ability to "understand" digital images and videos. "Understanding" corresponds to the transformation of visual images into descriptions of the world that are meaningful to thought processes and can prompt appropriate action. Computer vision is a field of Artificial Intelligence and has a wide variety of applications (face recognition, live translation of a text, autonomous vehicles...)

Computer vision started as early as the 1950s and distinguished from "rough" image processing by the desire to extract 3D representation from image. Recent resurgence in the field has been supported by considerable progress in machine learning and even more in deep learning. Deep learning algorithms have achieved accuracy close and in many application above, human performance on a set of benchmark tasks.

See [7] and [8] for more details.

Market potential [9] estimates the market size of computer vision to reach $48 billion in 2022. This size is expected to continue to grow given that computer vision has (and is expected to have even more in the future) a large range of industrial applications. Automatic inspection of production (in manufacturing), event detection (e.g. wild fire), object modeling (3D printing), navigation (autonomous vehicle), information organization (automatic labeling/organization of databases), etc.

Even though modern computer vision has already found many industrial use cases, the recent domination of deep learning methods [10] promises additional extension to a number of industrial applications in the coming years.

S1.2.4 Genome Editing

A brief description Genome editing (or genome engineering), is a type of genetic engineering in which DNA is inserted, deleted, modified or replaced in the genome of a living organism. Unlike early genetic engineering techniques that randomly inserts genetic material into a host genome, genome editing targets the insertions to site specific locations.

Genome editing was pioneered in the 1990s, its use was limited by low efficiencies of editing but has rapidly evolved in the 2000s. The three competing technologies in the field are zinc fingers, TALENs and CRISPR-Cas9. As described by [11], researchers initially relied on zinc fingers, a class of enzymes, in order to accurately edit genomes. However, such enzymes were rather expensive. In 2012, CRISPR-Cas9 (or simply CRISPR) was introduced. It relies on an enzyme called Cas9 that uses a guide RNA molecule to home in on its target DNA, then edits the DNA to disrupt genes or insert desired sequences. In addition to being more efficient and easy to use, it is also much cheaper than previous technologies, including TALENs, the third competing method. As an order of magnitude, CRISPR costs about 150 times less than zinc fingers. It is now widely used and a very active subject of research and invention.

See [11, 12, 13] for more details.

Market potential Market specialist Market and Markets projects the market size of genome editing at $11.7 billion by 2026. With countries moving to adjust the regulation to favor the development of genome editing applications, the growth of this technology is likely to be very high [14].

Indeed, genome editing is expected to have a large impact in gene therapy in general, either by replacing existing treatments or treating illness which could not be cured so far (e.g. Down syndrome). Genome engineering is also said to have the potential to eradicate diseases by disrupting the genes encoding the production of a virus receptor surface (e.g. HIV, herpes and hepatitis B) or by removing disease predisposition genes (e.g. cancer).

S1.2.5 Hydrogen Storage

A brief description Hydrogen energy storage denotes a set of technologies aiming at storing dihydrogen (H_2), in any form for later use. Traditionally, hydrogen generation is done by electrolysis using surplus energy production from renewable energy. The resulting hydrogen is then either used on-site or compressed and stored in tanks for transport and later use. However, recent interest in using hydrogen for energy storage on board clean transportation vehicles has led to the development of new storage methods that are safer, smaller and more easily integrated to mobile units.

Hydrogen is an interesting source of energy: it has the highest energy per mass of any fuel and its combustion does not generate CO2. Another interesting feature is that, unlike electricity, hydrogen can be stored for extended period of time. It is however rather inefficient in terms of energy per unit of volume, in particular due to its very low boiling points (20.3K or -253°C). It is therefore very important to develop advanced storage methods that have potential for higher energy density.

The most important existing hydrogen storage methods include physical storage methods based on either compression or cooling or a combination of the two (hybrid storage). More

recently, the use of nanomaterials has been proposed as an alternative option. Carbonaceous materials are currently being considered for onboard storage systems due to their versatility, multifunctionality, mechanical properties and low cost with respect to alternatives. The introduction of nanomaterials in onboard hydrogen storage systems is viewed as a major turning point for the future of hydrogen storage for the automotive industry.

For more details, see energy.gov

Market potential Various recent estimations of the market potential and future development of hydrogen storage are available. While the numbers vary, most experts concur that this technology should continue to grow in the next years.

Market analysis specialists such as Market Data Forecast or Allied Market Research forecast aggregate sales ranging from 19 to 25 billion dollars in 2027. The Hydrogen Council, a consortium of firms with stakes in the hydrogen market, project that "total investments will reach more than $300 billion in spending through 2030" [15]. Similarly, another group of market players, The Energy Transitions Commission, claimed that to reach zero net emission by 2050, an investment of $80 billion per annum will be required between 2020 and 2050 "for hydrogen production facilities and transportation & storage" [16].

S1.2.6 Self-driving Vehicle

A brief description A self-driving vehicle (or autonomous vehicle) is a vehicle that is capable of sensing its environment and moving safely with little or no human input. The technology can be divided into 2 broad sectors. First automated vehicle platform: items/hardware (e.g. sensors) and proceedings/software (e.g. algorithms) enabling the vehicle to make autonomous decisions. Second, smart environment which enables vehicles to interact with each other and their surrounding. Cars are classified into six different levels of autonomy. From no autonomy at all (level 0) to total autonomy, which makes human driving commands optional (level 5).

Since their diffusion in the early 20th century, cars have become progressively more and more autonomous. However, as of 2020, only a marginal number of products have reached level 3 (vehicle that can be driven with no need for human action, except in some specific cases which requires some level of attention). Waymo, Aptiv and Dena have developed such "robotaxis" but they are only deployed in a well-known extended neighborhood and under standard weather conditions. In December 2020, Waymo opened its service to the public, becoming the world's first robo-taxi service. Similarly, the Tesla autopilot requires constant attention from a human driver but in October 2020, full self driving beta mode was introduced with the ability to navigate previously unseen streets (not only high-speed lanes) in autonomous mode.

Large scale adoption of a fully autonomous vehicle would require important legal, insurance and infrastructure adjustments as well as important guarantees in terms of security, even if the technology is well advanced.

See [17] for more details.

Market potential For the reasons explained above, self driving vehicles are virtually unavailable on the market but might appear in the coming years with potentially already existing cars "transiting" to self-driving vehicles as software and regulations get updated.

The diffusion of this technology could impact many aspect of society. In addition to converting driving time into leisure, self driving vehicles could open up to a “car as a service” model rather than “ownership” model generating potential savings. Car could also become a non depreciating asset as updates of the car software and modularity could generate continuous improvement of existing cars.

References

[1] EPO. Patents and additive manufacturing: Trends in 3d printing technologies. Report, European Patent Office, 2020.

[2] Mark Zastrow. 3d printing gets bigger, faster and stronger. *Nature*, 578(7793):20–24, 2020.

[3] Sunny King and Scott Nadal. Ppcoin: Peer-to-peer crypto-currency with proof-of-stake. *self-published paper, August*, 19(1), 2012.

[4] Zibin Zheng, Shaoan Xie, Hongning Dai, Xiangping Chen, and Huaimin Wang. An overview of blockchain technology: Architecture, consensus, and future trends. In *2017 IEEE international congress on big data (BigData congress)*, pages 557–564. Ieee, 2017.

[5] Zibin Zheng, Shaoan Xie, Hong-Ning Dai, Xiangping Chen, and Huaimin Wang. Blockchain challenges and opportunities: A survey. *International journal of web and grid services*, 14(4):352–375, 2018.

[6] Brant Carson, Giulio Romanelli, Patricia Walsh, and Askhat Zhumaev. Blockchain beyond the hype: What is the strategic business value? Mckinsey insights, 2017.

[7] Athanasios Voulodimos, Nikolaos Doulamis, Anastasios Doulamis, and Eftychios Protopapadakis. Deep learning for computer vision: A brief review. *Computational intelligence and neuroscience*, 2018, 2018.

[8] Rostyslav Demush. A brief history of computer vision (and convolutional neural networks). https://hackernoon.com/a-brief-history-of-computer-vision-and-convolutional-neural-networks-8f 2019. Accessed: 2021-05-26.

[9] B Marr. Amazing examples of computer and machine vision in practice. *Forbes*, 2019.

[10] Andrej Karpathy, George Toderici, Sanketh Shetty, Thomas Leung, Rahul Sukthankar, and Li Fei-Fei. Large-scale video classification with convolutional neural networks. In *Proceedings of the IEEE conference on Computer Vision and Pattern Recognition*, pages 1725–1732, 2014.

[11] Heidi Ledford. Crispr, the disruptor. *Nature News*, 522(7554):20, 2015.

[12] J Travis. Crispr genome-editing technology shows its power. *Science*, 350:1456–7, 2015.

[13] Jon Cohen. How the battle lines over crispr were drawn. *Science*, 15, 2017.

[14] Stuart J Smyth and Justus Wesseler. The future of genome editing innovations in the eu. *Trends in Biotechnology*, 2021.

[15] Hydrogen Council. A perspective on hydrogen investment, market development and cost competitiveness. Hydrogen insight, 2021.

[16] Energy Transitions Commission. Making clean electrification possible: 30 years to electrify the global economy. Technical report, 2021.

[17] EPO. Patents and self-driving vehicles. the inventions behind automated driving. Report, EPO, 2018.
