# Supplementary material for: Identifying technology clusters based on automated patent landscaping
Source: PLoS One. 2023 Dec 14;18(12):e0295587. doi: 10.1371/journal.pone.0295587 (PMC10721033; doi:10.1371/journal.pone.0295587)
Supplement: S2 Appendix — (PDF) [file pone.0295587.s002.pdf]

S2 Construction of the seed

S2.1 Annotation guidelines

In order to manually assign one of the candidate patents to the seed or the anti-seed based on its abstract, we defined a series of tasks corresponding to each technologies. These tasks are presented in Table S2-1.

Table S2-1: Annotation guidelines

Technology	Options
**Additive Manufacturing**	- Create 3D printable model with computer aided design<br>- Examine stereolithography file for errors and inconsistency<br>- Convert model into a series of thin layers<br>- Manufacture materials for 3D printings<br>- Print 3D model
**Blockchain**	- Record transactions between two parties<br>- Serve as public transaction ledger of cryptocurrency<br>- Execute or enforce smart contract<br>- Hash tree verification / Verify the authenticity of documents / Proof of work<br>- Analyse transactions in a distributed ledger<br>- Manage Identity System based on the concept of peer-to-peer protocols (IDMS) / Mediate user authentication
**Computer Vision**	- Process digital images<br>- Analyse digital images<br>- Understand digital images
**Genome Editing**	- Target DNA sequence<br>- Break DNA sequence<br>- Edit DNA sequence
**Hydrogen Storage**	- Hydrogen production and compression<br>- Generate power from hydrogen gas<br>- Design vessel containment that is resistant to hydrogen permeation and corrosion (+ thermal management)<br>- Manufacture fuel cell using hydrogen<br>- Provide hydrogen to a hydrogen-powered device (fill, tank)
**Self-driving Vehicle**	- Enable vehicles to make autonomous decisions<br>- Automate vehicle handling<br>- Vehicle-to-vehicle communication<br>- Communication between vehicle and rest-of-the-world

Notes: Human annotator accepts or rejects a candidate patent depending on whether the patent's abstract clearly discusses one or more of the options listed.

S2.2 List of sources

In this section, we list the sources that we used to select relevant keywords, technological classes and patents to build the seed.

- **Additive Manufacturing:** [1, 2, 3, 4]
- **Blockchain:** [5, 6, 7, 8]
- **Computer Vision:** [9], [10] and [11]
- **Genome Editing:** [12]
- **Hydrogen Storage:** [13] and [14]
- **Self-driving Vehicle:** [15, 16]

S2.3 Criteria

We now detail the criteria by type and technology. The selection of candidate patents that we manually review to include in the seed must match at least one of the following criteria: 1) the patent's abstract contains at least one of the keywords (or keyphrases) listed in Section S2.3.1; 2) the patent's CPC codes include at least one code listed in Section S2.3.2; 3) the patent is highly similar to a patent listed Section S2.3.3. The latter patents are patents known to be at the core of the technology and the similarity is based on Google Patents embedding and are directly provided by Google Patent.

S2.3.1 Keywords

Additive Manufacturing 3d-printing, stereolithography, additive manufacturing, three-dimensional objects, rapid prototyping, additive material manufacturing three dimensional printing material, 3d-printing materials photolithography, fuse deposition mode

Blockchain blockchain, digital mining, bitcoin, cryptocoin, cryptocurrency, digital wallet, ethereum, smart contracts, record keeping, distributed ledger, distributed node, private ledger, public ledger, intelligent node, full node, digital signatures, public key, user identity, hashing, consensus methodologies, proof of work, proof of stake, deposition based, ripple

Computer Vision adaboost, xgboost, bayesian network, decision tree, genetic algorithm, gradient tree boosting, logistic regression, random forest, rankboost, support vector machine, multilayer perceptron, hidden markov model, generalized adversarial network, backpropagation, stochastic gradient descent, supervised training, reinforcement learning, neural network, self learning, semi supervised learning, unsupervised training, transfer learning, overfitting, active learning, clustering, data mining, deep learning, expert system, embedding, machine learning, fuzzy logic, feature selection, objective function, target function, regression model,

signal processing, computer vision, machine vision, lidar, character recognition, optical character recognition, handwritten character recognition, image to text, text recognition, face recognition, facial recognition, biometric data, biometrics, mass surveillance, face unlock, traffic cameras, object detection, edge detection, obstacle avoidance, motion tracking

Genome Editing dna editing, gene editing, genome engineering, recombinant targeting vectors, homologous recombination, double-strand dna break, homology-directed repair, targeted dna sequence, dna cleavage, fok1, sequence-specific nuclease system, zinc finger nuclease, cys2-his2, transcriptional activator-like effector nuclease, talens, clustered regularly interspaced short palindromic repeat, crispr/cas, cas9, pre-crrna, tracrrna, enzyme rnase, single guide rna, crispr-cpf1, ngago, single-stranded dna-guided argonaute endonuclease, natronobacterium gregoryi argonaute

Hydrogen Storage hydrogen fuel cells, hydrogen storage, liquid hydrogen, solid-state hydrogen storage, compressed hydrogen storage, dehydrogenation reaction, hydrogen gas, hydrogen fuel, hydrogen storage materials, hydrogen-powered device

Self Driving Vehicle self-driving vehicle, autopilot, driverless vehicle, autonomous vehicle, automated vehicles, vehicle connectivity, vehicle-to-vehicle communication, fleet management, vehicle lidar, vehicle sonar, vehicle radar, vehicle camera, object detection, obstacle detection, object classification, cruise control, pedestrian detection, environment mapping, surround view, blind spot detection, park assistance, lane departure, traffic sign recognition, drive assist system, trajectory generation, reactive control, path trajectory planning, manoeuvers planning

S2.3.2 CPC classes

Additive Manufacturing B81C2201/0184, G05B2219/49002, G05B2219/49003, G05B2219/49004, G05B2219/49005, G05B2219/49006, G05B2219/49007, G05B2219/49008, G05B2219/49009, G05B2219/49011, G05B2219/49013, G05B2219/49014, G05B2219/49015, G05B2219/49016, G05B2219/49017, G05B2219/49018, G05B2219/49019, G05B2219/49021, G05B2219/49022, G05B2219/49023, G05B2219/49024, G05B2219/49025, G05B2219/49026, G05B2219/49027, G05B2219/49028, G05B2219/49029, G05B2219/49031, G05B2219/49032, G05B2219/49033, G05B2219/49034, G05B2219/49035, G05B2219/49036, G05B2219/49037, G05B2219/49038, G05B2219/49039, A43D2200/60, A23P2020/253, B29C64/10, C08L101/00, B29C67/00, B22F3/00, G05B2219/49013, G03F7/70416, B28B1/001, B33Y10/00, B23K9/04, B23K10/027, B23K15/0086, B23K11/0013

Blockchain H04L009/08, H04L67/00, H04L009/10, H04L009/12, H04L009/14, H04L009/28, H04L29/06, G06Q20/00, G06F21/00, G06F12/14, G06Q20/06, G06Q20/10, G06Q20/20, G06Q20/32, G06Q20/36, H04L2209/00, G09C001/00, G09C001/02, G09C001/04, G09C001/06, H04L63/00, G06Q30/0619, G06F21/00, G06F021/24, G06F021/00, G06F021/02, G06F012/28, G06F012/14, G06F17/00

Computer Vision B25J9/161, G06F17/16, G06N5/003, G06N7/005, G06N7/046, B29C66/965, G08B29/186, F02D41/1405, G01N29/4481, G06F11/1476, G06F17/2282, H02P21/0014, H02P23/0018, H03H2222/04, Y10S128/924, Y10S128/925, B64G2001/247, F05B2270/707, F05B2270/709, F05D2270/709, G10H2250/151, H04L25/03165, H04Q2213/054, H04Q2213/343, B60G2600/1876, B60G2600/1878, B60G2600/1879, E21B2041/0028, F16H2061/0081, F16H2061/0084, G06F2207/4824, G10K2210/3024, G10K2210/3038, H03H2017/0208, B29C2945/76979, G05B2219/33002, G06T2207/20081 G06T2207/20084, G06T2207/20084, H04L2025/03464, H04L2025/03554, H04Q2213/13343, B60W30/06, B60W30/10, B60W30/12, B60W30/14, B60W30/17, G06T9/002, G10L25/30, G06K7/1482, G06T3/4046, B62D15/0285

Genome Editing A01H4/00, A01K67/00, C12N/1500, C12N1/00, C12N5/00, C12N7/00C12Y, C12N5/10, C12Q1/68, C12Q1/70, G01N33/00, A61K48/00, A61K31/7088, C07K14/00

Hydrogen Storage Y02E60/30, Y02E60/32, Y02E60/321, Y02E60/322, Y02E60/324, Y02E60/325, Y02E60/327, Y02E60/328, Y02E60/34, Y02E60/36, Y02E60/362, Y02E60/364, Y02E60/366, Y02E60/368, B01D53/02, C01B3/00-58, F17C2221/012, C22C19/03, C22C22/00, C22C33/00, F25B17/12, H01M4/38, H01M8/06, F17C2221/012, F17C6/00, F17C5/02

Self Driving Vehicle G08G1/02, G08G1/0967, G08G1/0968, G01S7/003, G07B15/063, G07C5/00, G07C5/12, E01F, E01F9/00, E01F9/40, H04W36/00, H04W76/50, B61L3/00, G05D1/0011, G05D1/0027, G05D1/0287, G05D1/0297, G08G1/00, G08G1/01, G08G1/09, G08G1/0968, G08G1/127, G08G1/16, G08G1/164, G08G1/20, G01S13/93, G10S13/931, G01S15/88, G01S15/93, G01S17/88, G01S17/93, G07C5/00, G07C5/01, G07C5/02, G07C5/03, G07C5/04, G07C5/05, G07C5/06, G07C5/07, G07C5/08, E01F9/00, B60L2240/70, B61L25/00, G01S7/00, G01S13/00, G01S15/00, G01S17/00, G01S7/00, G01S7/02, G01S7/52, G01S13/00, G01S13/86, G01S13/87, G01S13/93, G01S15/00, G01S15/025, G01S15/87, G01S15/931, G01S17/00, G06K9/00, G05D1/00, G05D1/0257, B60W2420/52, B60Y2400/3017, B60R19/00, G01S17/023, G01S17/06, G01S17/87, G01S17/88, G01S17/936, G01S7/48, G01S2013/9332, B60W2420/52, G06T1/0007, G06T1/0014, G06T1/20, G06K9/00362, G06K9/00785, G06K9/00791, H04N5/335, B60Y2400/3015, B60W2420/42, B60S1/56, G01C21/00, G01C21/26, G01C21/34, G01S7/52, G01S15/00, G05D1/00, G05D1/0027, G05D1/0088, G05D1/021, G05D1/0212, G05D1/0276, G05D1/0287, G05D1/02, G06T1/0007, G06T1/0014, G06T1/20, G08G1/16, G08G1/161, G08G1/22, H04W4/44, H04W4/46, F16D2500/31, B60L2240/60, B60L2240/62, B60W30/16, B60W2050/008, B60W2550/402, B60W2550/408 B60G17/015, B60G17/016, B60G17/0195, B60G2800/00, B60K28/04, B60W30/00, B60W40/00, F16D2500/508, G05D1/0088, G05D2201/0212, B60W30/095, B60W50/0097, G05D1/0212

S2.3.3 Representative patents

Additive Manufacturing US-4575330-A, US-5534104-A, US-6259962-A, US-5204055-A, US-5182056-A, DE-102013205724-A1, FR-3070302-B1, US-10076875-B2, US-8349239-B2, CN-108868141-A, CN-105569344-A, CN-105604327-A, WO-2018229418-A1, KR-101706473-B1, WO-2016111879-A1, US-20180141274-A1, WO-2008061909-A2, US-20170251713-A1, EP-1352619-B1, EP-3319545-B1, EP-3151782-B1, US-10441426-B2, US-9056017-B2

Blockchain EP-3125489-B1, US-9785369-B1, DE-102016104478-A1, US-9853819-B2, US-9842216-B2, US-9855785-B1, US-20180137465-A1, US-9635000-B1, EP-329562-A1, EP-3295350-B1, CN-105719172-A, CN-105701372-B, US-9836908-B2, US-9818092-B2, US-9824031-B1, US-10643202-B2, CN-105844505-A, US-9298806-B1, CN-105790954-B, US-9858781-B1, US-9853977-B1, US-9641338-B2, US-9641342-B2, EP-325719-B1

Computer Vision US-8953886-B2, WO-2003023696-A1, US-5881172-A, US-20170024607-A1, US-20170169205-A1, US-20170169303-A1, US-20170235931-A1, US-20200175326-A1, US-10872228-B1

Genome Editing WO-2000041566-A9, WO-2003087341-A3, WO-2010079430-A1, WO-2011072246-A2, US-8440431-B2, US-8440432-B2, US-8450471-B2, US-8566363-B2, WO-2014093661-A2, WO-2013176772-A1, US-20170367280-A1

Hydrogen Storage US-20080248355-A1, CN-1322266-C, US-7678362-B2, US-7118611-B2, CN-203500844-U, US-7094493-B2, US-10622655-B2, WO-2019239141-A1, US-8871671-B2, JP-6061354-B2, EP-2554694-B1, FR-2939784-A1, CA-2980664-C, CN-103797142-A, US-7678479-B2, JP-6418680-B2, DE-102009016475-B4, US-7093626-B2, DE-102013203892-A, KR-101107633-B1, JP-4849775-B2, JP-3706611-B2, US-6875536-B2, JP-5338903-B2

Self Driving Vehicle US-20050088318-A1, US-9293045-B2, US-9723457-B2, US-10405215-B2, WO-2019052353-A1, US-10089537-B2, US-10564639-B1, DE-112019000049-T5, US-20190384304-A1, DE-112019000122-T5, US-20170030728-A1, US-20190265703-A1, WO-2019094843-A1

References

[1] EPO. Patents and additive manufacturing: Trends in 3d printing technologies. Report, European Patent Office, 2020.

[2] Aalt van de Kuilen. Using patbase for patent landscaping: a case study on 3d printing techniques. Report, Minesoft, 2015.

[3] Priya Anish Mathews, Swati Koonisetty, Sanjay Bhardwaj, Papiya Biswas, Roy Johnson, and G Padmanabham. Patent trends in additive manufacturing of ceramic materials. *Handbook of Advanced Ceramics and Composites: Defense, Security, Aerospace and Energy Applications*, pages 319–354, 2020.

[4] Mark Zastrow. 3d printing gets bigger, faster and stronger. *Nature*, 578(7793):20–24, 2020.

[5] IIPRD. Sample patent landscape study - blockchain. Report, IIPRD, 2017.

[6] IP Australia. Blockchain innovation: A patent analytics report. Report, IP Australia, 2018.

[7] Nigel S Clarke, Björn Jürgens, and Victor Herrero-Solana. Blockchain patent landscaping: An expert based methodology and search query. *World Patent Information*, 61:101964, 2020.

[8] Thomas Isaacson. The blockchain patent landscape shows accelerating growth. Ipwatchdog articles, IPwatchdog, 2020.

[9] WIPO. Data collection method and clustering scheme. Technical report, 2019.

[10] WIPO. Artificial intelligence. Technical Report 2019: artificial intelligence, 2019.

[11] Zhang Bo, Lyu Lucheng, Wang Yanpeng, Zhao Yajuan, and Qian Li. Global patent analysis of computer vision. *Science Focus*, 16(2):72–83, 2021.

[12] Osmat Azzam Jefferson, Simon Lang, Kenny Williams, Deniz Koellhofer, Aaron Ballagh, Ben Warren, Bernard Schellberg, Roshan Sharma, and Richard Jefferson. Mapping crispr-cas9 public and commercial innovation using the lens institutional toolkit. *Transgenic Research*, 30(4):585–599, 2021.

[13] Manuel Baumann, Tobias Domnik, Martina Haase, Christina Wulf, Philip Emmerich, Christine Rösch, Petra Zapp, Tobias Naegler, and Marcel Weil. Comparative patent analysis for the identification of global research trends for the case of battery storage, hydrogen and bioenergy. *Technological forecasting and social change*, 165:120505, 2021.

[14] Intellectual Patent Office. *Low-carbon hydrogen: A worldwide overview of patenting related to the UK's ten point plan for a Green Industrial Revolution*. 2021.

[15] EPO. Patents and self-driving vehicles. the inventions behind automated driving. Report, EPO, 2018.

[16] Rico Lee-Ting Cho, John S Liu, and Mei Hsiu-Ching Ho. The development of autonomous driving technology: perspectives from patent citation analysis. *Transport Reviews*, 41(5): 685–711, 2021.
