# Supplementary material for: Identifying technology clusters based on automated patent landscaping
Source: PLoS One. 2023 Dec 14;18(12):e0295587. doi: 10.1371/journal.pone.0295587 (PMC10721033; doi:10.1371/journal.pone.0295587)
Supplement: S3 Appendix — (PDF) [file pone.0295587.s003.pdf]

S3 Additional results

Figure S3-1: Share of patent families with an abstract in English

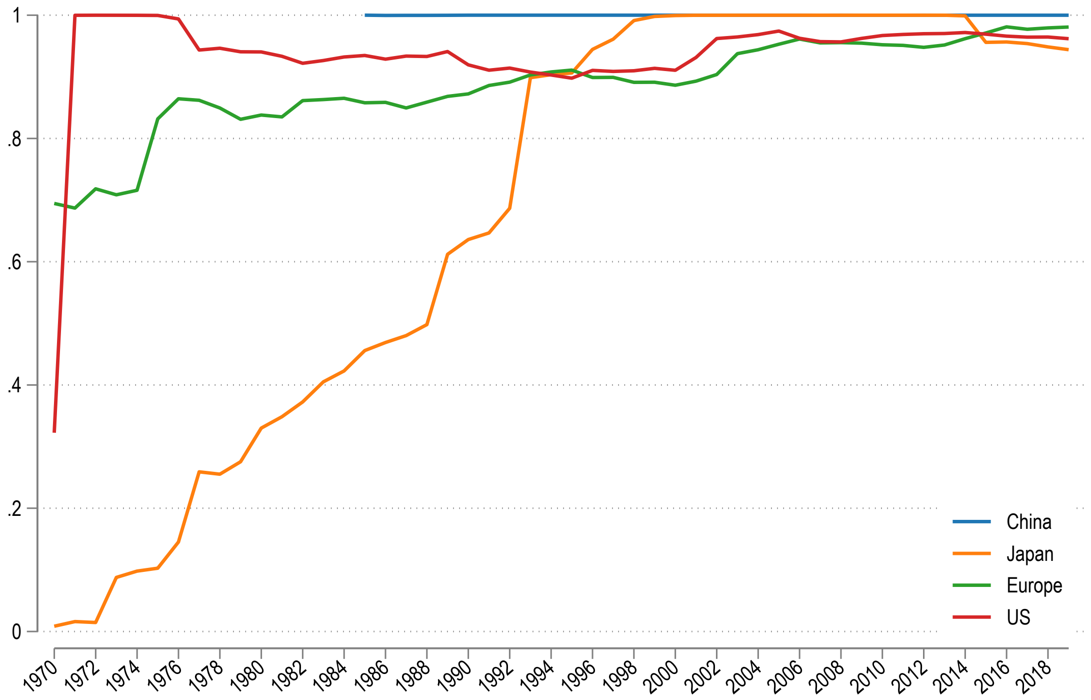

Notes: Share of patents with at least one patent in the same family with an abstract in English in each of the four patent offices: USPTO (US), CNIPA (CN), EPO and European national patent offices (EP) and JPO (JP). The year of publication is reported in x-axis. European patent offices include the European Patent Office, as well as national patent offices of all EU countries, UK, Norway and Switzerland.

Figure S3-2: Number of patent publications by technology

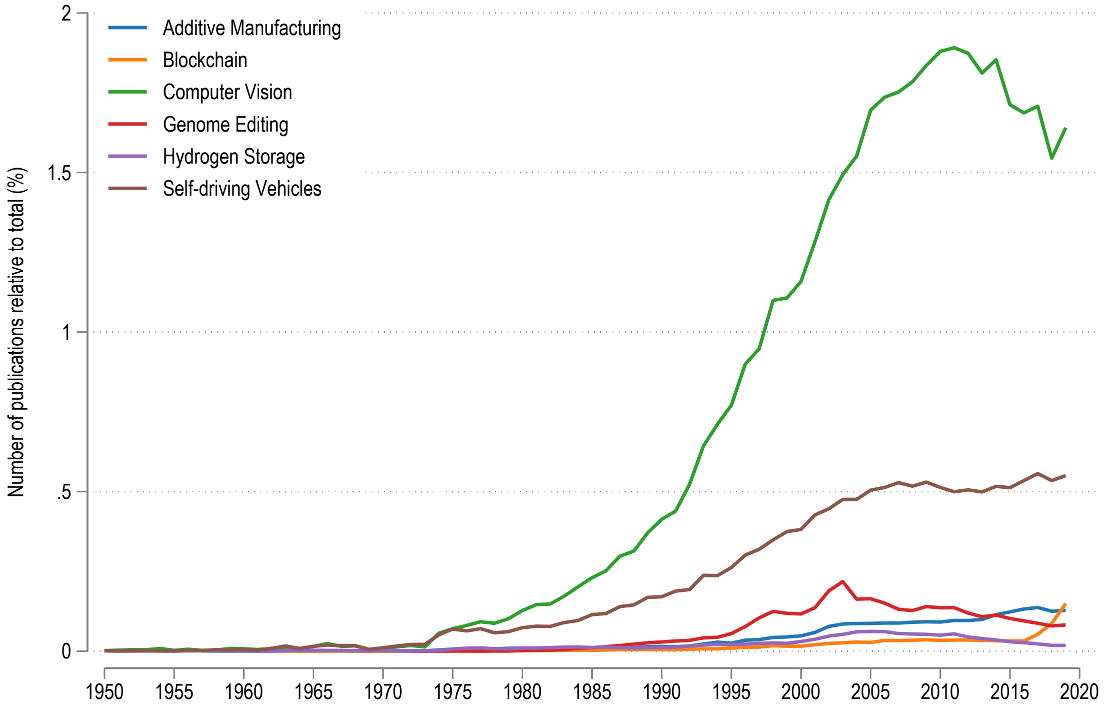

Notes: Total number of patents published yearly at the US, Chinese, European and Japanese patent offices in each of the six technologies considered. This number has been standardized by the total number of patents published in these patents office in any technology. The year of publication is reported in x-axis. European patent offices include the European Patent Office, as well as national patent offices of all EU countries, UK, Norway and Switzerland.

Table S3-1: Precision from simple rule-based classification

	**Additive Manufacturing**	**Blockchain**	**Computer Vision**
**CPC**	0.34 (224)	0.01 (458)	0.26 (298)
**Keywords**	0.16 (218)	0.09 (456)	0.20 (254)
**Patents**	0.02 (42)	0.09 (53)	0.4 (5)
	**Genome Editing**	**Hydrogen Storage**	**Self driving Vehicle**
**CPC**	0.05 (172)	0.14 (211)	0.12 (222)
**Keywords**	0.89 (158)	0.24 (221)	0.36 (239)
**Patents**	0.57 (7)	0.16 (32)	0.42 (24)

Notes: Precision computed on the test set - unseen during training using simple rule-based classification (either using only CPC, only keywords or similar patents from the set of manually added patents). Number in parentheses corresponds to the number of patents.

Table S3-2: 10 most cited articles by technology

	Additive manufacturing	Blockchain	Computer vision	Genome editing	Hydrogen storage	Self driving vehicle
1	10.1116/1.1412895	10.1145/121133.121137	10.1109/tcsvt.2003.815165	10.1038/35078107	10.1016/s0378-7753(97)02724-9	10.4271/961010
2	10.1021/ma60060a028	10.1145/37499.37500	10.1109/cvpr.2001.990517	10.1093/emboj/20.23.6877	10.1002/ceat.270100130	10.3189/s0260305500002822
3	10.1007/bfb0053286	10.1145/50202.50214	10.1109/iccv.2001.937709	10.1101/gad.862301	10.1016/s0360-3199(00)00021-5	10.1049/cp:19960454
4	10.1109/vlsit.2007.4339708	10.1109/tcsvt.2003.815173	10.1109/tcsvt.2003.815173	10.1038/35888	10.2172/460349	10.1109/ivs.2000.898318
5	10.1145/237170.237191	10.1109/tcsvt.2003.815165	10.1109/iccv.1999.790410	10.1126/science.2315699	10.1002/cjce.5450690503	10.1109/imtc.2001.929558
6	10.1145/311535.311556	10.1109/2.16	10.1007/bfb0053007	10.1126/science.1231143	10.1002/cjce.5450690504	10.1109/irds.2002.1041378
7	10.1145/37402.37422	10.1109/tcsvt.2003.814963	10.1016/b978-0-08-051581-6.50024-6	10.1126/science.1232033	10.1246/cl.1993.41	10.1109/imtc.1999.776736
8	10.1116/1.591000	10.1109/dcc.1998.672152	10.1109/34.888718	10.1038/332323a0	10.1016/0360-3199(95)00131-x	10.1049/cp:19980155
9	10.1117/12.968423	10.1145/989.990	10.1109/tcsvt.2007.905532	10.1038/327070a0	10.1016/s0378-7753(97)02760-2	10.1007/s10967-005-0069-2
10	10.1002/pol.1979.170170410	10.1109/tcsvt.2003.815175	10.1889/1.3256703	10.1038/313810a0	10.1016/s0378-7753(97)02796-1	10.1016/s0034-6667(99)00031-7

Notes: Top 10 academic papers cited in the patents in each six technologies in the frontpage retrieved using PatCit [1].

References

[1] Gaétan de Rassenfosse and Cyril Verluise. PatCit: A Comprehensive Dataset of Patent Citations, March 2020. URL `https://doi.org/10.5281/zenodo.3710994`.
