# Supplementary figures and images for: Identifying technology clusters based on automated patent landscaping
Source: PLoS One. 2023 Dec 14;18(12):e0295587. doi: 10.1371/journal.pone.0295587 (PMC10721033; doi:10.1371/journal.pone.0295587)

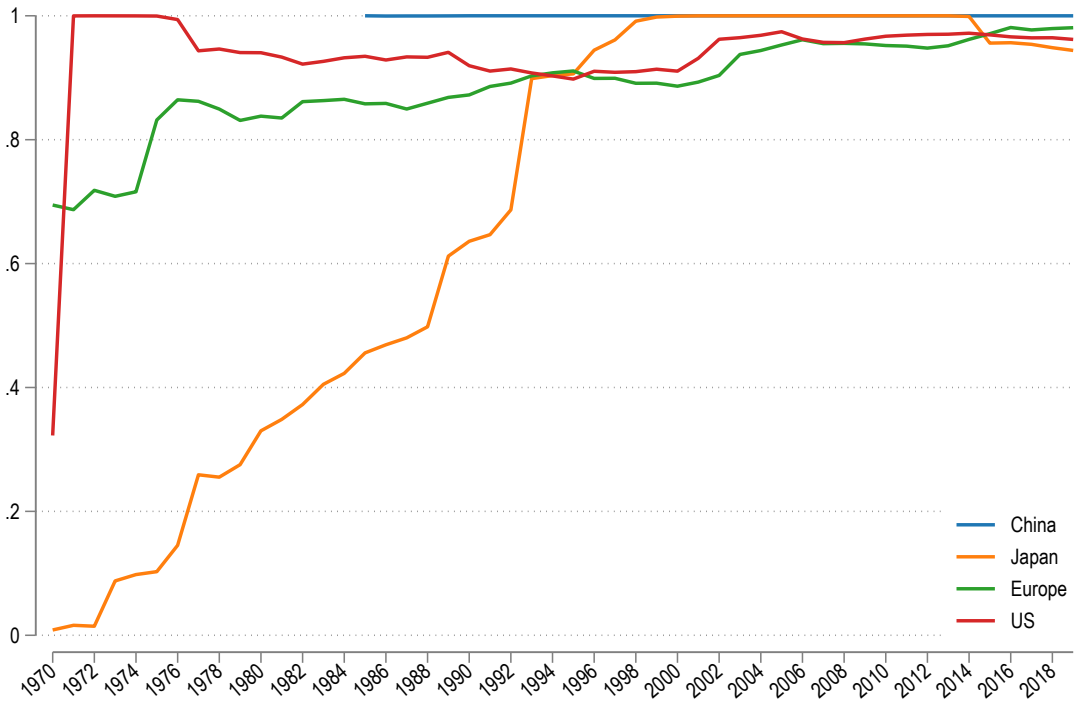

Supplement: S1 Fig — (PDF) [file pone.0295587.s004.pdf]

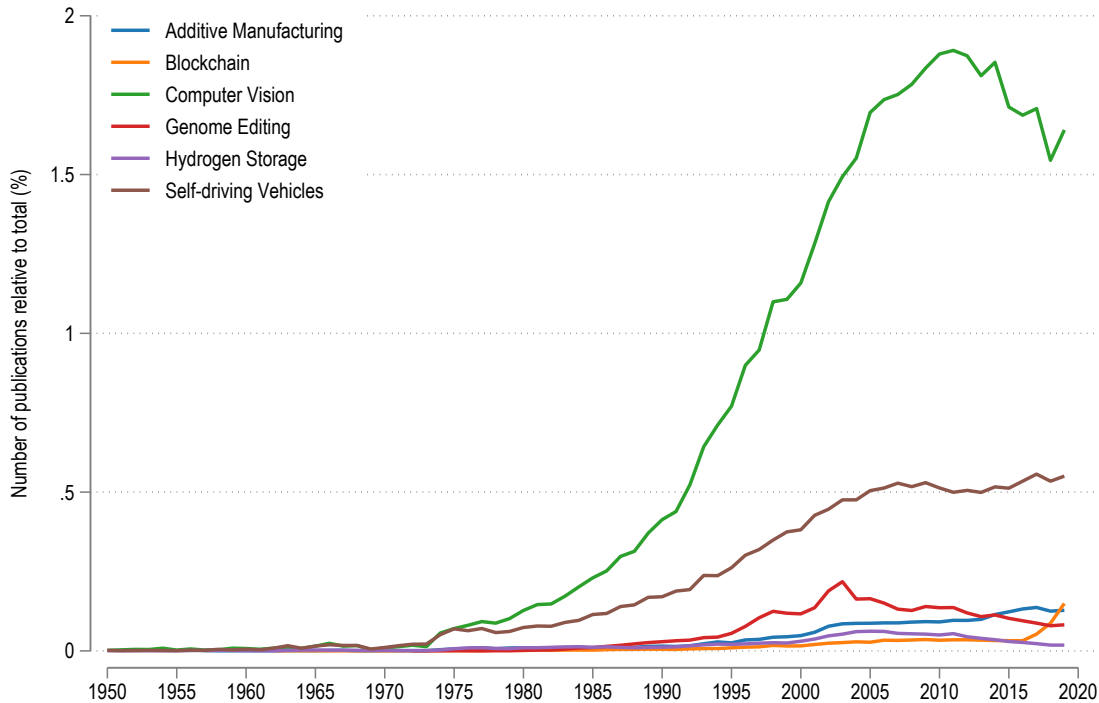

Supplement: S2 Fig — (PDF) [file pone.0295587.s005.pdf]
